# Highly efficient preparation of active *S*-phenyl-L-cysteine with tryptophan synthase using a chemoenzymatic method

**DOI:** 10.1186/s12896-019-0538-2

**Published:** 2019-07-18

**Authors:** Lisheng Xu, Xingtao Zhang, Guizhen Gao, Sun Yue

**Affiliations:** Department of Life and Food Science, Suzhou University, Suzhou, 234000 China

**Keywords:** *S*-phenyl-L-cysteine, Tryptophan synthase, Chemoenzymatic

## Abstract

**Background:**

S-Phenyl-L-cysteine is regarded as having potential applicability as an antiretroviral/protease inhibitor for human immunodeficiency virus (HIV). In the present study, optically active *S*-phenyl-L-cysteine was prepared in a highly efficient manner from inexpensive bromobenzene using tryptophan synthase through a chemoenzymatic method.

**Results:**

The chemoenzymatic method used a four-step reaction sequence. The process started with the reaction of magnesium and bromobenzene, followed by a Grignard reaction, and then hydrolysis and enzymatic synthesis using tryptophan synthase. Through this approach, *S*-phenyl-L-cysteine was chemoenzymatically synthesized using tryptophan synthase from thiophenol and L-serine as the starting material.

**Conclusions:**

High-purity, optically active *S*-phenyl-L-cysteine was efficiently and inexpensively obtained in a total yield of 81.3% (> 99.9% purity).

## Background

S-Phenyl-L-cysteine exhibits the dual advantages of showing long-term effects and having a chemical configuration that is comparable to the anti-AIDS drug nelfinavir. The possibility that S-phenyl-L-cysteine can, like nelfinavir, act as an effective suppressant of HIV protease [1], has increased the importance of developing more tractable approaches for producing these chemical compounds. This is further underscored by the potential for the synthesis of phenyl-L-cysteine and its use in multiple biological activation mechanisms [2–4].

The potential utility of optically active *S*-phenyl-L-cysteine has inspired pharmaceutical chemical scientists to explore new and effective routes to its synthesis. To date, however, only a few synthetic methods have emerged for the preparation of *S*-phenyl-L-cysteine. Previously, *S*-phenyl-L-cysteine was prepared using tryptophan synthase in *Escherichia coli* MT-10242 and *Neurospora crassa* ATCC 14692. The reaction time for preparing *S*-phenyl-L-cysteine using these strategies was 15 h [5, 6], and thus, they were inefficient based on their reaction time requirements. In another case, *S*-phenyl-L-cysteine was prepared by reacting L-cysteine hydrochloride and a soluble single-valent copper (Cu) salt with the diazonium salt of phenylamine [7, 8]. The yields of *S*-bromo phenyl-L-cysteine from this copper-mediated reaction, however, were not impressive (37%). A subsequent effort to synthesize *S*-phenyl-L-cysteine from *S*-bromo phenyl-L-cysteine and mercapturic acid was successful [9, 10].

**Fig. 1:**
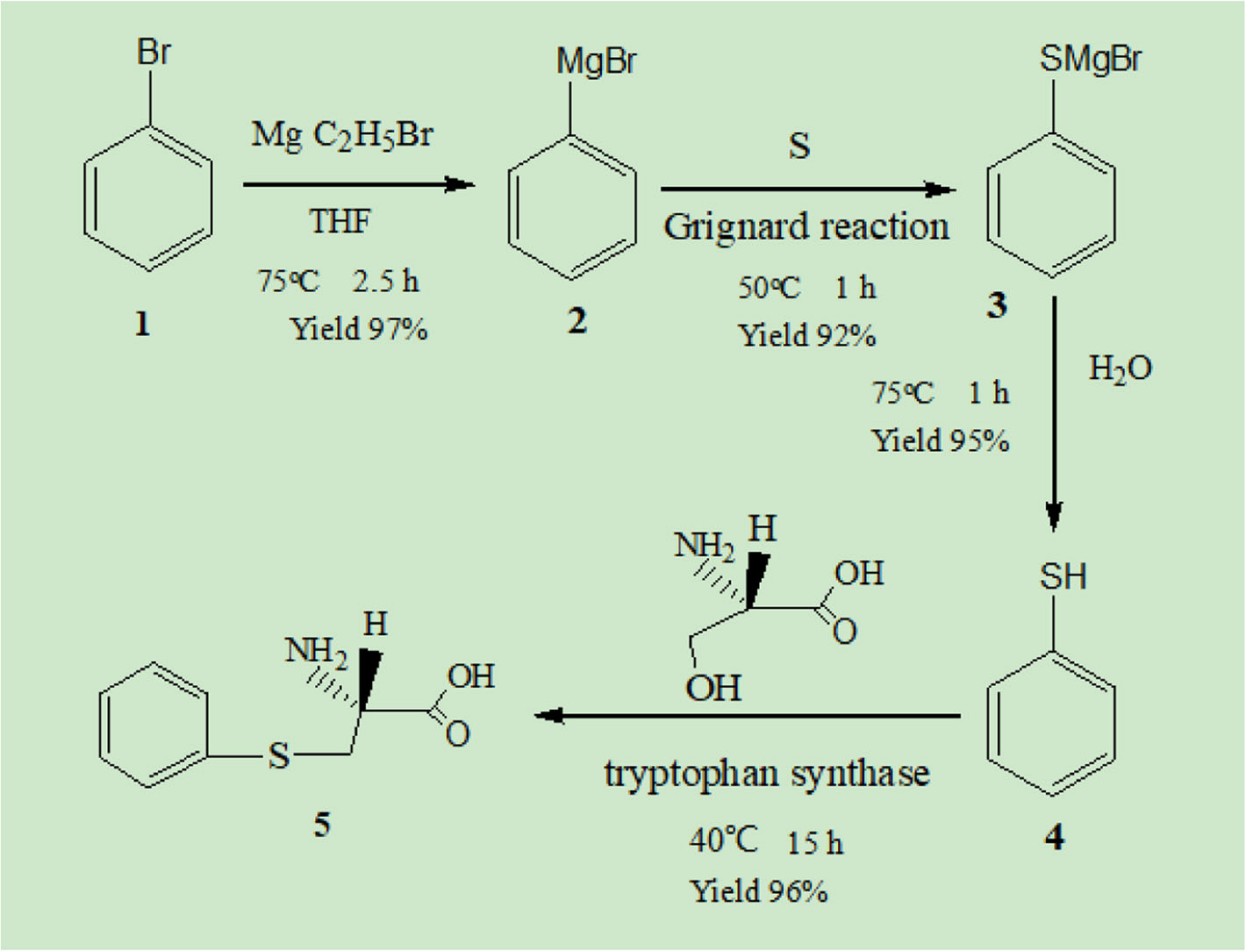
Chemoenzymatic preparation of optically active *S*-phenyl-L-cysteine

In the present study, *S*-phenyl-L-cysteine was synthesized from thiophenol and L-serine using a recombinant tryptophan synthase (E.C. 4.2.1.20) obtained from *E. coli* k-12 MG1655. This approach resulted in high yields of optically active *S*-phenyl-L-cysteine (5) (Fig. 1). The products from the reaction of thiobenzyl alcohol and ethanethiol were then isolated to demonstrate the enzymatic synthesis of the corresponding S-substituted L-cysteines [11]. Our findings indicate that the tryptophan synthase from *E. coli* k-12 MG1655 effectively catalyses the synthesis of L-cysteine from L-serine and sodium hydrosulfide [12].

## Results

### Preparation of phenylmagnesium bromide (2)

Phenylmagnesium bromide was prepared using magnesium and bromobenzene. Phenyl magnesium bromide was separated through filtration (2) (yield: 97%). ^1^H NMR (400 MHz, CDCl_3_): δ 7.01~7.66 (m, 5H) Combustion elemental analysis calculated (Anal. Calcd) for C_6_H_5_MgBr: C, 39.99; H, 2.78; Br, 43.88. Found: C, 39.96; H, 2.77; Br, 43.89. ESI-MS (m/z): 181.0121 [M + H]^+^. The calculated mass of phenyl magnesium bromide: 180.1141.

### Preparation of thiophenyl magnesium bromide (3)

Thiophenyl magnesium bromide was prepared by using sulfur and phenyl magnesium bromide. Thiophenyl magnesium bromide (3) was formed after cooling (yield: 92%). ^1^H NMR (400 MHz, CDCl_3_): δ 7.29–7.32 (m, 5H). Anal. Calcd for C_6_H_5_SMgBr: C, 33.96; H, 2.35; S, 15.09; Br, 37.27. Found: C, 33.94; H, 2.34; S, 15.11; Br, 37.26. ESI-MS (m/z): 213.0311 [M + H]^+^. The calculated mass of phenyl magnesium bromide: 212.7812.

### Preparation of thiophenol (4)

Thiophenol (4) was isolated by distillation of the upper layer of the solution. Thiophenol (4) was obtained in 95% yield. ^1^H NMR (400 MHz, CDCl_3_): δ 6.97~7.42 (m, 5H), 3.40 (s, 1H) Anal. Calcd for C_6_H_6_S: C, 65.45; H, 5.45; S, 29.10. Found: C, 65.44; H, 5.49; S, 29.07. ESI-MS (m/z): 111.1821 [M + H^]+^. The calculated mass of thiophenol: 111.0172.

### Preparation of *S*-phenyl-L-cysteine (5)

**Fig. 2:**
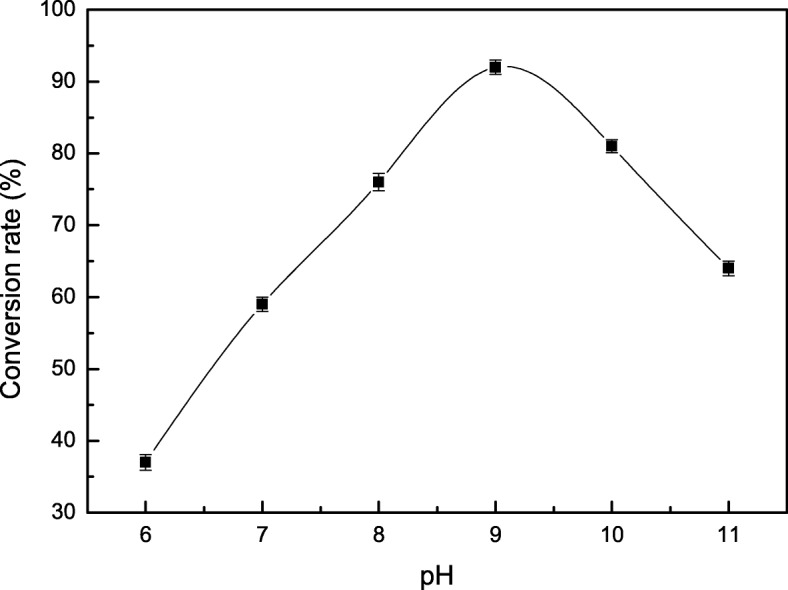
Effect of pH on the tryptophan synthase-catalysed synthesis of S-phenyl-L-cysteine. Tryptophan synthase was directly mixed with the substrate (180 mmol/L) at 40 °C for 14 h using Trion X-100 at 0.02%

**Fig. 3:**
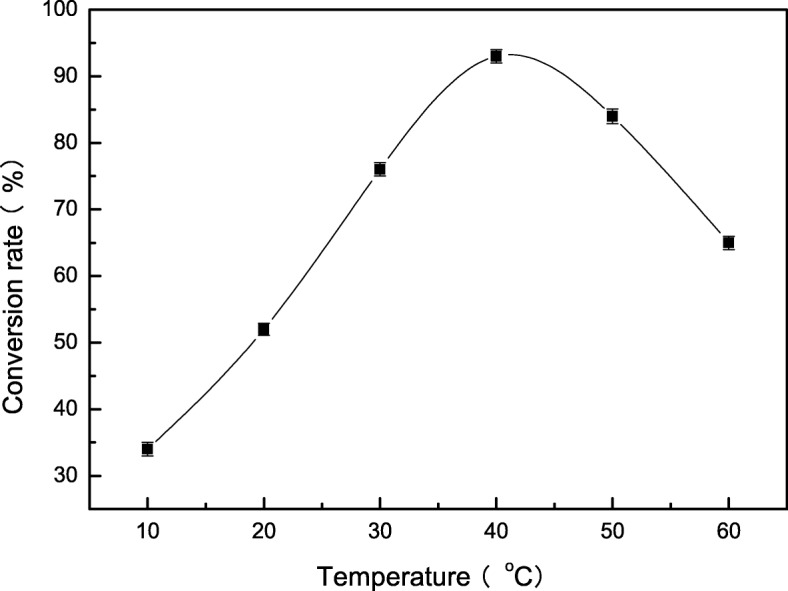
Effect of temperature on the tryptophan synthase-catalysed synthesis of S-phenyl-L-cysteine. Tryptophan synthase was directly mixed with the substrate (180 mmol/L) at pH 9.0 for 14 h using Trion X-100 at 0.02%

**Fig. 4:**
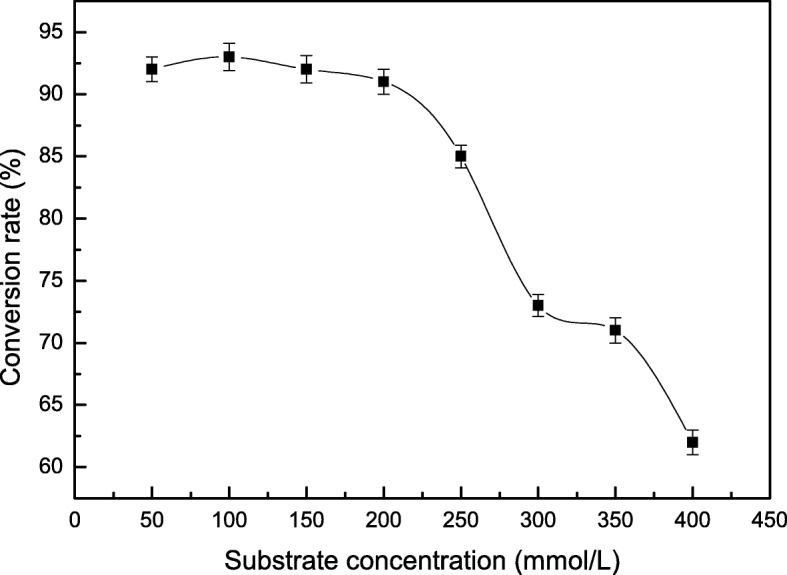
Effect of substrate concentration on the tryptophan synthase-catalysed synthesis of S-phenyl-L-cysteine. Tryptophan synthase was directly mixed with the substrate at 40 °C and pH 9.0 for 14 h using Trion X-100 at 0.02%

**Fig. 5:**
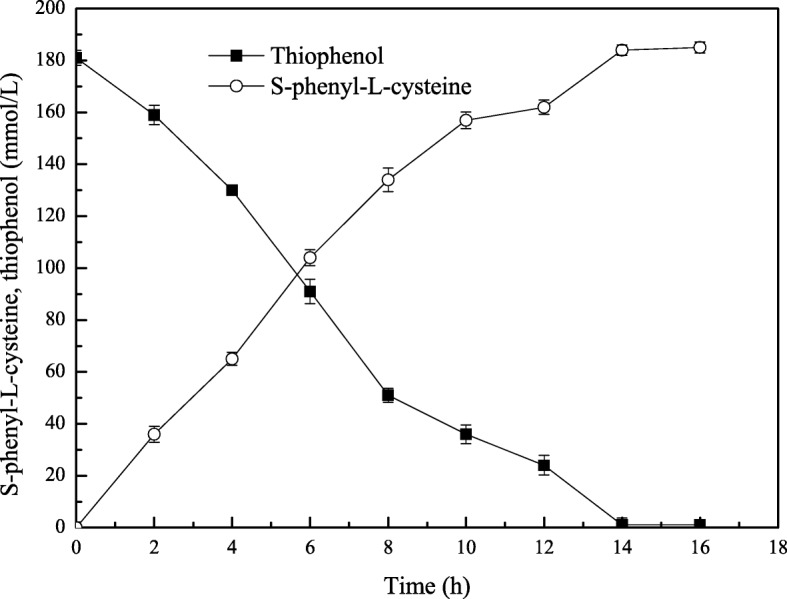
Changes in the concentrations of *S*-phenyl-L-cysteine and thiophenol. The concentrations of *S*-phenyl-L-cysteine (o) and phenylthiol alcohol (■) were measured at different times

**Fig. 6:**
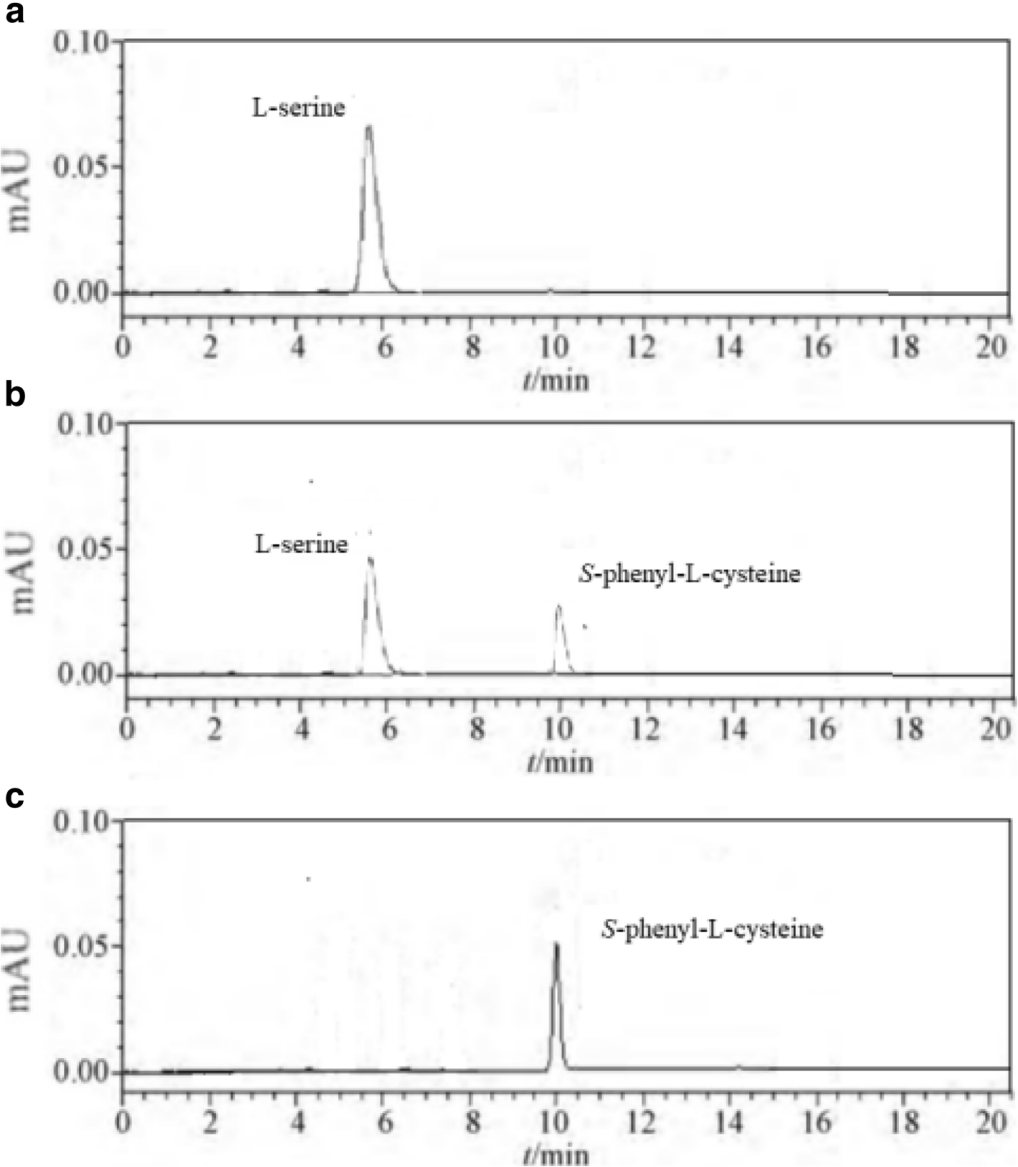
HPLC chromatograms of S-phenyl-L-cysteine. (Reaction times a: 0 h, b: 6 h, and c: 8 h)

**Fig. 7:**
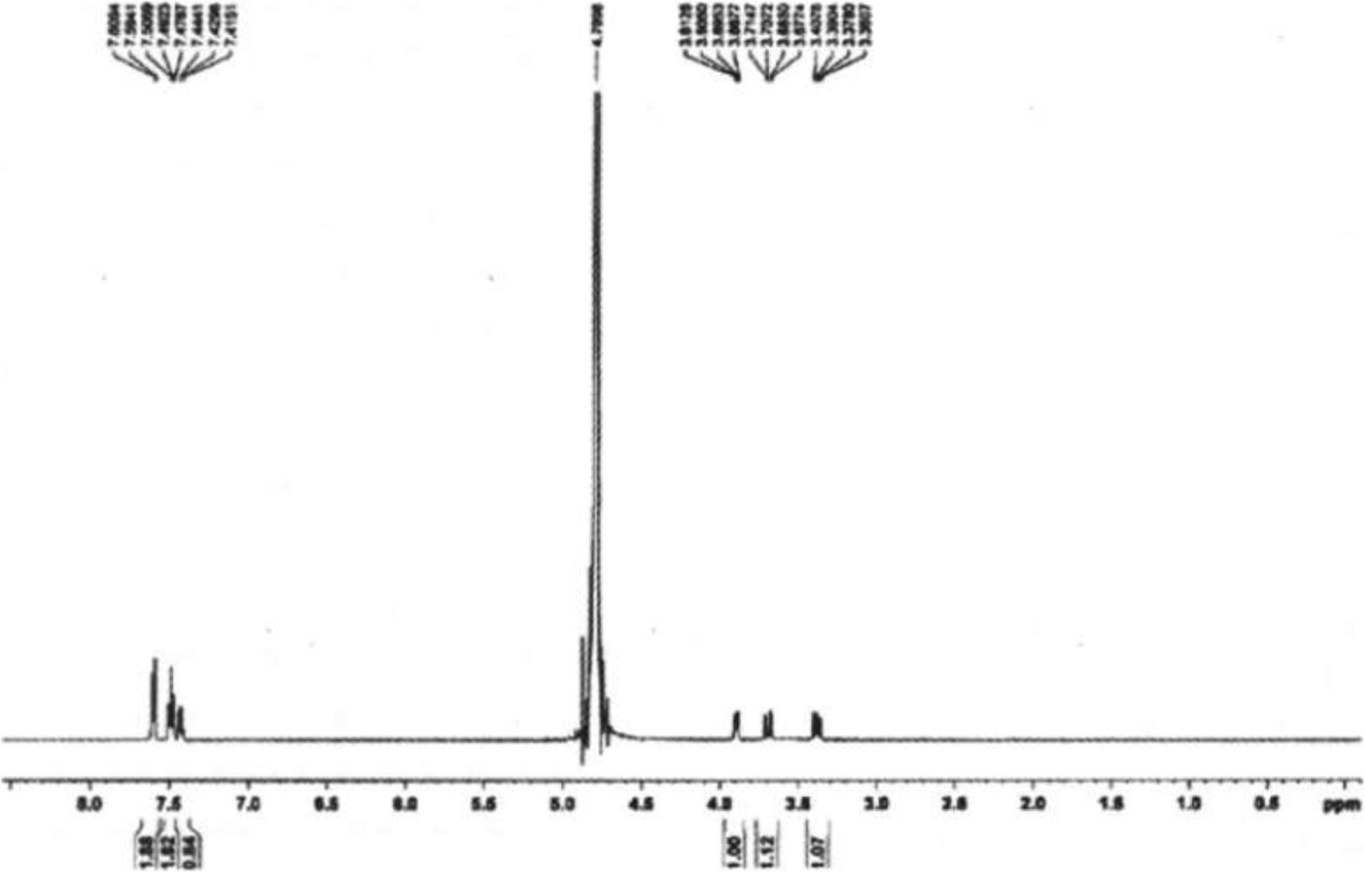
^1^H NMR spectrum of S-phenyl-L-cysteine (400 MHz, D_2_O)

**Fig. 8:**
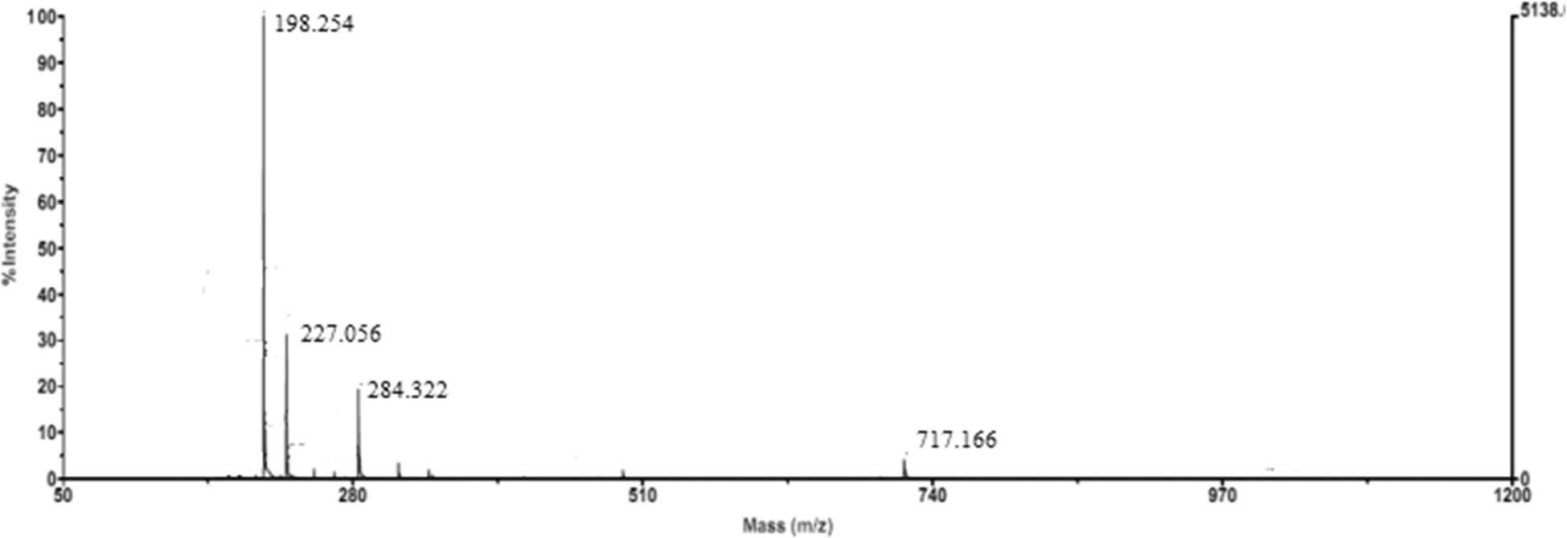
Mass spectrum of S-phenyl-L-cysteine

The activity of tryptophan synthase is dependent on factors such as substrate concentration, temperature, and pH. Tryptophan synthase was directly mixed with substrate (180 mmol/L) at pH values from 6 to 11 at 40 °C, and the reactions were allowed to proceed for 14 h. We found the optimal initial pH for the synthesis of S-phenyl-L-cysteine was 9.0 (Fig. 2). The effect of temperatures from 10 °C to 60 °C on S-phenyl-L-cysteine synthesis was investigated. The best yield of S-phenyl-L-cysteine was achieved at 40 °C (Fig. 3). The effect of substrate concentrations from 50 mmol/L to 400 mmol/L on S-phenyl-L-cysteine synthesis was investigated. The optimal substrate concentration was 180 mmol/L (Fig. 4). Tryptophan synthase was directly mixed with thiophenol and L-serine under the optimum reaction conditions of pH 9.0, 40 °C, using Trion X-100 at 0.02% (Fig. 5). After drying the crystals, 16.04 g of *S*-phenyl-L-cysteine was obtained (yield: 96%). The purity of S-phenyl-L-cysteine was 99.9% as verified by HPLC (Fig. 6). Specific rotation [α] $\overset{20}{D}$ = + 73~ + 75 ° (*c* = 1, 1.5 M H_2_SO_4_). ^1^H NMR (400 MHz, D_2_O): δ 2.81 (dd, J = 7.82, 3.13 Hz, H), 2.88 (d, J = 3.13 Hz, H), 3.13(d, J = 7.82 Hz, H), 7.21~7.39 (m, PhH, 5H) (Fig. 7). Anal. Calcd for C_9_H_11_NO_2_S: C, 54.75; H, 5.57; N, 7.09. Found: C, 54.72; H, 5.52; N, 7.05. ESI-MS (m/z): 198.254 [M + H]^+^ (Fig. 8). The calculated mass of S-phenyl-L-cysteine: 197.2312.

## Discussion

Tryptophan is a naturally occurring amino acid that is synthesized by tryptophan synthase in plants and microorganisms. While indole-3-glycerol phosphate has been synthesized by the α-subunit of tryptophan synthase, it has not been used for the synthesis of tryptophan analogues. A series of analogues of L-tryptophan were synthesized through a β-reaction using tryptophan synthase [13]. Likewise, we reported the synthesis of *S*-phenyl-L-cysteine using tryptophan synthase from L-serine derived from the hydrolysis of keratin from industrial wastewater and thiophenol [14]. High-purity tryptophan was produced in excellent yield.

Thiophenols are synthesized from phenols through a reaction with thiophosgene to form an aryl chlorothionoformate [15] that is then reacted with hydrogen sulfide over an absorptive catalyst, such as activated carbon or calcined petroleum coke [16]. The production of a thiophenol involves the reaction of hydrogen sulfide and a source of hydrogen with a halogenated aromatic compound [17]. All of the above methods require heating at a high temperature and have high production costs.

In this study, thiophenol was formed by a simple, 4-step sequence involving the reaction of magnesium and bromobenzene (1) to form phenylmagnesium bromide (2), a Grignard reaction with sulfur to form thiophenyl magnesium bromide (3), and hydrolysis using sulfuric acid to form the resulting thiophenol (4).

## Conclusions

Optically active *S*-phenyl-L-cysteine (5) was synthesized using tryptophan synthase from low-cost bromobenzene (1). Tryptophan synthase was successfully applied for the preparation of optically active *S*-phenyl-L-cysteine in excellent purity (> 99.9%) and high yield (81.3%).

## Methods

### Reagents and instruments

All chemical reagents were of analytical grade and were purchased from Aladdin Reagent Corporation (Shanghai, China). The chemical compositions of *S*-phenyl-L-cysteine and thiophenol were verified using HPLC (Shimadzu High-Performance Liquid Chromatograph, Kyoto, Japan). ^1^H NMR spectra were recorded on a Bruker DRX500 (500 MHz; Tokyo, Japan). Optical rotations were recorded using a WZZ-2B polarimeter (Hinotek, Ningbo, China). Mass spectra were recorded on a Mariner ESI-TOF mass spectrometer (Applied Biosystems, Foster City, CA). The enantiomeric purities of the residual substrate were determined as described by Zheng [18]. Elemental compositions were measured using a trace element auto analyser (EA3000 type).

### Enzymes

**Fig. 9:**
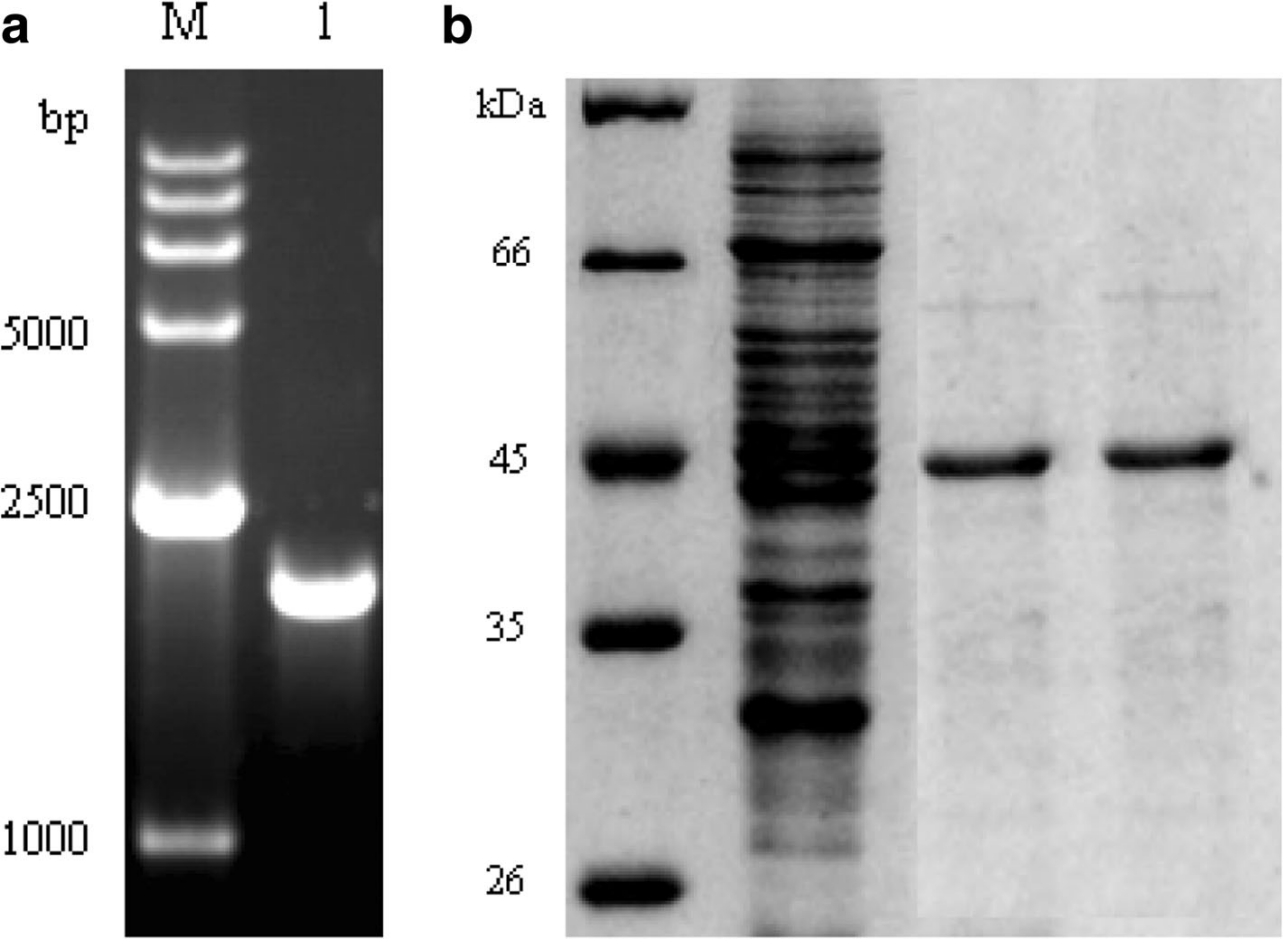
**a**. Agarose gel electrophoresis analysis of tryptophan synthase gene. Lane M: nucleic acid molecular mass standards. Lane 1: the product of tryptophan synthase PCR. **b**. SDS-PAGE analysis of recombinant *E. coli* expressing recombinant tryptophan synthase

The gene encoding tryptophan synthase was cloned from *E. coli* k-12 MG1655. The *E. coli* strain BL21(DE3) carrying the recombinant plasmid Duet-trpBA (DM206) was constructed in our laboratory (Fig. 9). Tryptophan synthase appeared as an intense protein band with an apparent molecular mass of approximately 45 kDa. The amplified PCR product was separated by agarose gel electrophoresis. The gene encoding tryptophan synthase was 2 kb. A loopful of strain culture was used to inoculate 40 ml of LB broth in a 200 ml Erlenmeyer flask. The flask was incubated at 30 °C for 12 h on a rotary shaker at 170 rpm. Tryptophan synthase was purified according to the protocol described by Tsunehiko et al. [19]. The cells containing tryptophan synthase were placed in 40 ml of 40 mM Tris-HCl (pH 7.5) and then ultrasonicated at 4 °C. The cell-free extracts of tryptophan synthase were then fractionated using (NH_4_)_2_SO_4_ (20–60%). The active fractions of tryptophan synthase were collected by (NH_4_)_2_SO_4_ sedimentation; they were then applied to a DEAE-Sepharose CL-6B column (4.5× 85 cm; Seikagaku Kogyo, Japan) and equilibrated with 45 mM Tris-HCl (pH 7.5) containing 1 mM manganese sulfate. The tryptophan synthase was eluted using 45 mM Tris-HCl (pH 7.5) containing 1 mM manganese sulfate. The final tryptophan synthase sample was found to be homogeneous based on SDS-polyacrylamide gel electrophoresis. Following the addition of (NH_4_)_2_SO_4_ to the final tryptophan synthase sample, it was stored at 4 °C in buffer containing 45 mM Tris-HCl (pH 7.5) with 1 mM manganese sulfate, which is the buffer system that had been used throughout the tryptophan synthase purification procedure. The activity of tryptophan synthase was determined by the conversion rate of *S*-phenyl-L-cysteine. Tryptophan synthase was directly mixed with the substrate (100 mmol/L) at 40 °C and pH 9.0 for 14 h using Trion X-100 at 0.02%.

### Preparation of thiophenol (4)

The preparation of thiophenol included three steps: (1) the reaction of magnesium and bromobenzene, which formed phenyl magnesium bromide; (2) a Grignard reaction with sulfur, which formed thiophenyl magnesium bromide; and (3) the hydrolysation of thiophenyl magnesium bromide by sulfuric acid, which afforded thiophenol (4).

For the first step, bromobenzene was dissolved in THF. Bromobenzene (25%) was added to the reactor containing magnesium. Bromobenzene (15.6 g) was dissolved in tetrahydrofuran (THF, 50 mL). A quarter of the bromobenzene solution and the magnesium (3.07 g) were added to the reactor. The reactor was stirred at 170 rpm under N_2_. Bromoethane (1.66 g) in THF (5 mL) was added into the reactor. The mixture was heated at 75 °C. The remaining bromobenzene in THF was dropped into the reactor after cooling. The reactor was stirred at 75 °C for 150 min. After cooling the filtrate to 30 °C, the formed phenyl magnesium bromide (2) was separated by filtration.

For the second step, sulfur (4.05 g) was dropped into the reactor with phenyl magnesium bromide (2). The reactor was stirred at 50 °C. Thereafter, the mixture was heated at reflux for 1 h. Thiophenyl magnesium bromide (3) was formed upon cooling.

In the third step, thiophenyl magnesium bromide was hydrolysed by sulfuric acid. Sulfuric acid (44 ml; 2.27 M) was dropped into the reactor over 20 min at 60 °C. The reactor was stirred at 75 °C for an additional 60 min. Another portion of sulfuric acid (14.4 ml; 6.93 M) was dropped into the reactor over 20 min. The resulting solution formed two layers.

### The enzymatic step

In this study, thiophenol (4) was prepared through three simple steps (Fig. 1). *S*-Phenyl-L-cysteine (5) was synthesized using tryptophan synthase. In the biosynthetic reaction, the reactor was stirred at 170 rpm under N_2_. The content of thiophenol gradually decreased as *S*-phenyl-L-cysteine was formed over 14 h. After 14 h, HCl was added to the enzymatic reaction to adjust its pH to 0.5, generating S-phenyl-L-cysteine. The enzymatic reaction mixture was filtered, and the filtrated was adjusted to pH 2.5 using sodium hydroxide. After cooling the filtrate to 10 °C, the target product (*S*-phenyl-L-cysteine) was obtained.

## Data Availability

The datasets used and/or analysed in the current study are available from the corresponding author upon reasonable request.

## Abbreviations

*E. coli*: *Escherichia coli*
HIV: Human immunodeficiency virus
